# Oxidative stress and redox state-regulating enzymes have prognostic relevance in diffuse large B-cell lymphoma

**DOI:** 10.1186/2162-3619-1-2

**Published:** 2012-03-26

**Authors:** Pekka Peroja, Anna Kaisa Pasanen, Kirsi-Maria Haapasaari, Esa Jantunen, Ylermi Soini, Taina Turpeenniemi-Hujanen, Risto Bloigu, Laura Lilja, Outi Kuittinen, Peeter Karihtala

**Affiliations:** 1 Department of Oncology and Radiotherapy, University of Oulu and Oulu University Hospital, Oulu, Finland; 2 Department of Pathology, University of Oulu, Oulu, Finland; 3 Institute of Clinical Medicine and Department of Medicine, University of Eastern Finland and Kuopio University Hospital, Kuopio, Finland; 4 Department of Clinical Pathology and Forensic Medicine, School of Medicine, University of Eastern Finland, Cancer Center of Eastern Finland and Kuopio University Hospital, Kuopio, Finland; 5 Medical Informatics Group, University of Oulu, Oulu, Finland

**Keywords:** Antioxidant enzyme, Nitrotyrosine, Prognosis, Reactive oxygen species, Thioredoxin

## Abstract

**Background:**

Oxidative stress and redox-regulating enzymes may have roles both in lymphomagenesis and resistance to lymphoma therapy. Previous studies from the pre-rituximab era suggest that antioxidant enzyme expression is related to prognosis in diffuse large B-cell lymphoma (DLBCL), although these results cannot be extrapolated to patient populations undergoing modern treatment modalities. In this study we assessed expression of the oxidative stress markers 8-hydroxydeoxyguanosine (8-OHdG) and nitrotyrosine and the antioxidant enzymes thioredoxin (Trx), manganese superoxide dismutase (MnSOD) and glutamate-cysteine ligase (GCL) via immunohistochemistry in 106 patients with DLBCL. All patients were treated with CHOP-like therapy combined with rituximab. Immunostaining results were correlated with progression-free survival, disease-specific survival and traditional prognostic factors of DLBCL.

**Results:**

Strong 8-OHdG immunostaining intensity was associated with extranodal involvement (p = 0.00002), a high International Prognostic Index (p = 0.002) and strong Trx (p = 0.011) and GCL (p = 0.0003) expression. Strong Trx staining intensity was associated with poor progression-free survival (p = 0.046) and poor disease-specific survival (p = 0.015). Strong GCL immunostaining intensity predicted poor progression-free survival (p = 0.049). Patients with either strong Trx or strong nitrotyrosine expression showed significantly poorer progression-free survival (p = 0.003) and disease-specific survival (p = 0.031) compared with the other patients.

**Conclusions:**

The redox state-regulating enzymes GCL and Trx are promising markers in the evaluation of DLBCL prognosis in the era of modern immunochemotherapy.

## Background

Of lymphomas, diffuse large B-cell lymphoma (DLBCL) accounts for 30% to 40%. DLBCL comprises three different subtypes as determined by gene expression profiling: the germinal center (GC) type, the activated B-cell type and a third heterogeneous type that cannot be included in either of the two other categories [1]. In routine practice the subtypes are classified by means of by immunohistochemical grouping (Hans' algorithm) into GC and non-GC subtypes [2]. Standard treatment for DLBCL consists of CHOP (cyclophosphamide, doxorubicin, vincristine and prednisone) or CHOP-type regimens and rituximab. Rituximab has revolutionized the treatment of DLBCL by increasing survival in all International Prognostic Index (IPI) classes [3]. New molecular markers predicting the outcome of DLBCL are still important in order to optimize treatment.

Production of reactive oxygen species (ROS) is common to all aerobic cells, but excessive oxidative stress may lead to variable pathological conditions. The most important factor in the redox environment is the creation and elimination of ROS. These are important cellular mediators and their formation is strictly controlled under physiological circumstances. They are mostly formed in the mitochondria as a by-product of oxygen metabolism. The ROS generated in mitochondria plus, for example, hydrogen peroxide and superoxide, can affect cellular signaling pathways by activating signaling cascades or redox-sensitive transcriptional factors. In many pathological conditions, e.g. atherosclerosis, diabetes and cancer, ROS creation and elimination is out of control, leading to excessive amounts [4-8]. Antioxidants are important in the elimination of ROS, thus maintaining the normal physiological state [9].

Redox proteins can be used in the evaluation of oxidative stress. Manganese superoxide dismutase (MnSOD) is a mitochondrial enzyme and it is the most important superoxide dismutase in physiological conditions. Normally, MnSOD makes ROS less harmful by reducing superoxide anions to hydrogen peroxide, which is then neutralized to water and oxygen [10]. Thioredoxin (Trx) functions by reducing oxidized proteins by way of cysteine thiol-disulfide exchange and therefore it limits the damage caused by oxidative stress [11]. Gamma cysteine ligase (GCL) is a rate-limiting enzyme in the creation of glutathione (GSH). Glutathione reduces disulfide bonds and thus repairs oxidative damage by serving as an electron donor [12]. 8-Hydroxydeoxyguanosine (8-OHdG) and nitrotyrosine are markers of oxidative damage. 8-OHdG is an end-product of oxidative damage to DNA [13]. Nitrotyrosine in its free form and in proteins marks oxidative damage to protein structures [14]. Nitrotyrosine can also by itself cause DNA damage [15] and bring about apoptosis [16].

An oxidatively balanced redox status may affect carcinogenesis by modulating DNA in particular, but also other cellular structures [10]. On the other hand, oxidative stress mediates the effects of many cytostatic drugs by causing sublethal DNA damage and thus activating apoptosis. Oxidative stress markers are prognostically important, e.g. in breast cancer [17,18], ovarian carcinoma [19], head and neck squamous cell carcinoma [20] and acute myeloid leukemia [21]. The prognostic role of oxidative stress in DLBCL has been evaluated in two previous studies in the pre-rituximab era, with conflicting results. The present study was undertaken to evaluate the role of oxidative stress and counteracting enzymes in more detail in DLBCL patients treated with modern immunochemotherapy.

## Results

Strong cytoplasmic 8-OHdG staining intensity was associated with extranodal involvement (p = 0.00002) and a high IPI score (divided into two groups, 0-2 and 3-5) (p = 0.002). There were no other significant associations between clinical prognostic parameters and staining intensities of the studied markers. The staining intensities of Trx, GCL and nitrotyrosine correlated with the percentage of malignant positive cells. Intensities were discovered to be better suited to assess survival estimates. Staining patterns were discovered and analyzed in our previous study [22]. The staining intensity of cytoplasmic 8-OHdG correlated with high Trx (p = 0.011) and GCL (p = 0.0003) intensities. There were no other associations between the studied oxidative stress markers and antioxidant enzymes.

**Figure 1:**
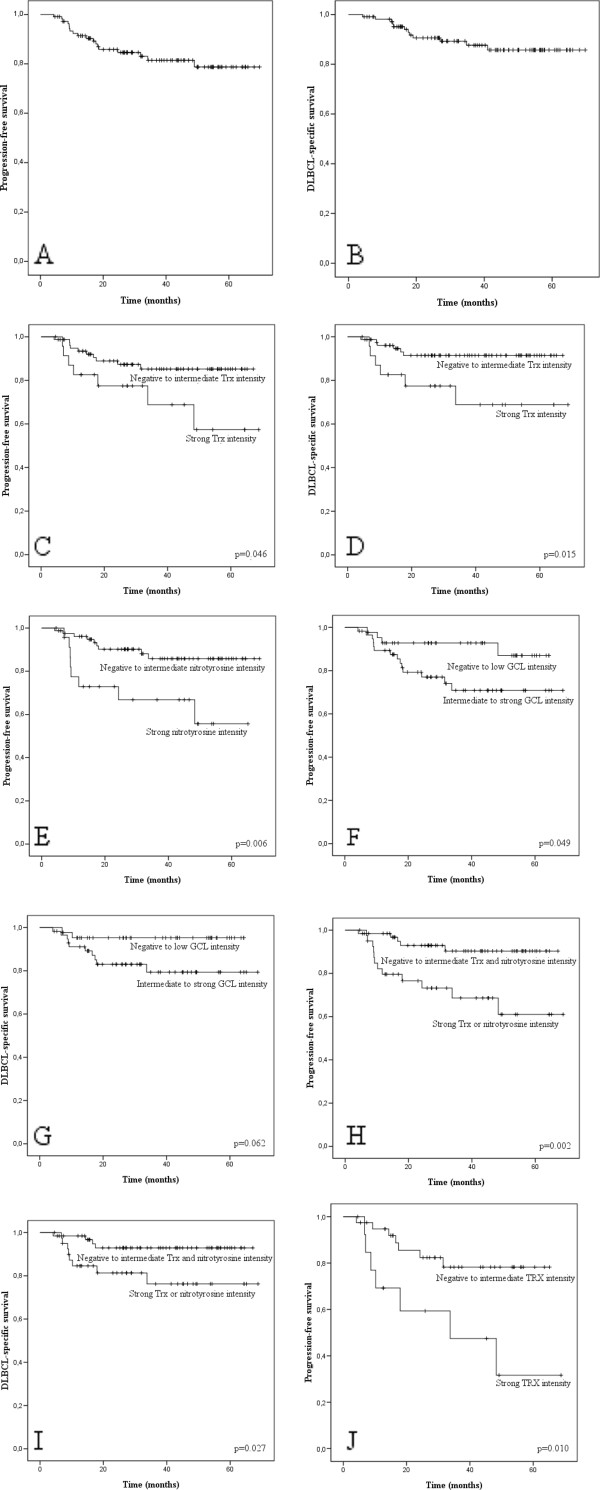
**Progression-free survival (A) and DLBCL-specific survival (B) of all patients**. Strong Trx intensity was associated with both poor progression-free (C) and disease-specific (D) survival. Nitrotyrosine as a marker of nitrosative stress was a marker of poor prognosis (E). GCL-positive patients had worse disease-specific survival (F) and a trend towards shorter disease-specific survival (G). Patients with strong nitrotyrosine and/or Trx expression had significantly worse relapse-free (H) and DLBCL-specific (I) survival than other patients. Trx intensity was a powerful prognostic indicator in the non-GC group (J), but not in patients with GC phenotype.

Overall 5-year progression-free survival was 81.4% and overall 5-year DLBCL-specific survival was 87.6%. Strong Trx staining intensity was associated with poor progression-free survival (p = 0.046) and poor disease-specific survival (p = 0.015) (Figure 1). The rate of 5-year progression-free survival was 85.1% in patients with negative to moderate Trx intensity and 57.4% in those with strong Trx intensity. Patients with strong cytoplasmic nitrotyrosine immunostaining had poor progression-free survival (p = 0.006; 5-year survival 85.8% in patients with negative to moderate staining and 55.7% in those with strong positivity). Moderate to strong GCL staining intensity was associated with worse prognosis, 5-year progression-free survival being 87.0% versus 70.8% (p = 0.049). Likewise, 5-year disease-specific survival was 94.6% (negative to light GCL immunostaining) versus 77.5% (moderate to strong GCL immunostaining) (p = 0.062). MnSOD and 8-OHdG staining results were not associated with progression-free or disease-specific survival.

Patients with either strong Trx or strong nitrotyrosine immunostaining intensities had significantly poorer progression-free survival (p = 0.003) and disease-specific survival (p = 0.031) compared with the other patients. In patients with a non-GC phenotype, strong Trx expression was associated with progression-free survival (p = 0.010); this was not observed in patients with a GC-phenotype. The rate of 5-year progression-free survival was 78.4% in patients with negative to moderate Trx immunostaining and it was 31.6% in those with strong Trx intensity in the non-GC phenotype group.

## Discussion

This is the first study in which the prognostic value of oxidative stress markers and antioxidant enzymes in DLBCL patients treated by means of modern immunochemotherapy has been assessed. We discovered that low immunoexpression of GCL, Trx and nitrotyrosine was associated with favorable progression-free survival and Trx was also significant as regards disease-specific survival. Intensive Trx immunostaining also revealed a high-risk group when only non-GC phenotype patients were included. Staining of GCL was almost significant as regards prediction of poor disease-specific survival. Our results suggest that excessive antioxidant and high nitrotyrosine expression are associated with adverse prognosis.

The prognostic role of oxidative stress in lymphomas has been studied previously, with conflicting results. A study by Tome *et al. *(2005) [23] concerned analysis of the levels of antioxidant enzymes and redox-associated proteins. Their results suggested that the group with the worst prognosis were those with decreased expression of antioxidant enzymes, including catalase, glutathione peroxidase and MnSOD, and increased expression of Trx. In a study by Andreadis *et al. *(2007) [24], the expression of genes in the glutathione family was examined. Their data suggested that overexpression of these genes correlated with worse prognosis. They used the redox signature score created by Tome *et al. *[23] and found that their results were exactly the opposite, i.e. overexpression of antioxidants and redox-state proteins correlated with poor prognosis. However, in neither of these studies had the patients received modern immunochemotherapy. Because prognostic markers are highly associated with the therapeutic modality used, the results cannot be extrapolated to patient populations undergoing modern treatments. We have previously carried out thorough evaluation of the expression of oxidative stress markers in different forms of lymphoma. In a recent study [22] we found that high levels of GCL and nitrotyrosine, and also low levels of MnSOD correlated with poor prognosis, but the number of patients with DLBCL in that study was only 18. The aim of the present study was to assess the relationships between antioxidant enzymes, oxidative stress markers and prognosis in cases of diffuse large B-cell lymphoma treated in the rituximab era.

At first glance the results reported by Andreadis *et al. *and Tome *et al. *seem to be contradictory, but when evaluating the data according to the expression of individual enzymes, the differences are not so striking. Comparing the results reported by Tome *et al. *and those in the current study, most are contradictory, as their data indicated that antioxidant function would be a cellular protector. Both our study and theirs showed that strong intensity of Trx was associated with worse prognosis. In the study by Andreadis *et al. *the authors came to the same conclusion as ourselves, i.e. that the GSH system is related to poor prognosis and excessive antioxidant expression worsens the prognosis. Because the biological functions are complex and antioxidant enzymes may have both positive and negative effects on malignant cell growth, the expression of these markers should probably be analyzed individually and also possibly disease-specifically. Redox state-regulating enzymes may have prognostic value regardless of treatment, since our results are largely in line with those in two previous studies where different treatments were applied. The expression of 8-OHdG, the most commonly used marker of oxidative DNA damage, is a significant prognostic factor in various solid malignancies, but in DLBCL it does not seem to have a prognostic role. Nevertheless, high cytoplasmic 8-OHdG expression, which reflects ROS-derived mitochondrial DNA adduct formation, was associated with features of aggressive disease such as high IPI class and extranodal involvement. Expression of 8-OHdG was associated with high Trx and GCL expression and this suggests therefore that these enzymes might be induced under heavy oxidative stress in DLBCL cells.

Rituximab has revolutionized DLBCL treatment and patient prognosis. However, some patients still succumb to their lymphomas. IPI scoring is at the moment the only clinically relevant prognostic scoring system. Results regarding the prognostic value of gene expression profiling-based grouping are contradictory [25,26]. Unfortunately, IPI scoring can no longer identify a patient population with a 5-year prognosis of less than 50%. Therefore, reliable biological prognostic factors and especially factors describing basic biological phenomena which might serve as predictors of treatment results are needed. Our results indicate that oxidative stress markers have correlations with prognosis in cases of DLBCL. Preclinical data indicate that GSH induces resistance against key lymphoma chemotherapeutic agents. *In vitro*, drugs such as indomethacin can counteract this adverse effect by reducing GSH levels [27]. This implies that drugs that suppress GSH might be therapeutically beneficial in lymphomas with high GSH expression. This may also have relevance to the Trx system [28]. Trx is an activator of nuclear factor κB (NF-κB), which inhibits apoptosis and increases proliferation [29]. Non-GC-type DLBCL is characterized by constitutional activation of NF-κB. It has been suggested that high levels of NF-κB may be a sign of adverse prognosis [30,31]. Our observation of strong Trx intensity and poor prognosis in non-GC phenotype patients might be explained by activation of NF-κB and its anti-apoptotic properties through Trx overexpression.

Resistance to doxorubicin [27] and vincristine [32] has been linked to increased amounts of intracellular GSH. Cyclophosphamide treatment depletes GSH and increases the amount of oxidative stress in human ovarian granulosa cells [33]. Therefore, it is plausible that an increased amount of GSH limits the effectiveness of cyclophosphamide and might be connected to lymphoma chemoresistance. Our results may partly be explained by activation of GCL, which increases the amount of GSH and may therefore bring about resistance to chemotherapy. Doxorubicin sensitivity has also been linked to an excessive amount of Trx [34].

## Conclusions

In conclusion, oxidative stress and cellular redox state-regulating enzymes seem to play an important role in DLBCL also in the era of modern therapies. They are of prognostic relevance and may participate in chemoresistance. These results should be authenticated in a prospective study and the role of chemoresistance should be studied in lymphoma cell culture models. If the results can be confirmed *in vitro *they probably should be taken into account in phase I-II clinical trials dealing with patients with antioxidant enzyme-overexpressing chemoresistant DLBCL.

## Methods

### Patient material

Table 1 summarizes the characteristics of the patients included in the study. There were 106 patients with histologically confirmed DLBCL from whom paraffin-embedded tissue sections from diagnostic lymph nodes, extralymphatic tumor site samples or coarse needle biopsy samples were available. Detailed patient information was collected in each case. The lymphomas were diagnosed and treated at Oulu University Hospital and Kuopio University Hospital between the years 2003-2009. Diagnoses were reviewed by an experienced hematopathologist. The diagnostic work-up included history and physical examination, blood chemistry, bone marrow biopsy and aspiration and whole body computer tomography. The median age of the patients was 64.6 years (21-90 yr). CHOP-like therapy combined with rituximab antibody treatment was given to all patients. Involved field radiotherapy was applied after immunochemotherapy at the sites of bulk disease with residual masses according to individual discretion. DLBCL phenotypes were divided into three groups according to Hans' algorithm: (i) a GCB phenotype with positive CD-10 and/or bcl-6 and negative MUM-1 immunostaining; (ii) a non-GCB phenotype with positive MUM-1 and positive or negative bcl-6 and negative CD-10 immunostaining; (iii) a third type consisting of cases with characteristics of both previous types [2]. The ethical committee of the Northern Ostrobothnia Hospital District has approved the study design (reference number 42/2010).

**Table 1:** Patient characteristics.

Median age	64.6
Male	52.8%

Stage	

I-II	46.3%

III-IV	53.7%

Primary therapy	

CEOP	17.6%

CHOP	63.0%

CHOEP	18.5%

CHOP + IMVP-16	0.9%

Radiotherapy	53.7%

Rituximab	100%

GC phenotype	33.3%

Non-GC phenotype	57.3%

B-symptoms	37.0%

LD high	64.8%

WHO > 1	19.4%

Extranodal involvement > 1	22.6%

IPI	

0-2	59.0%

3	18.1%

4-5	22.8%

CHOP, cyclophosphamide, doxorubicin, vincristine and prednisone; CEOP, cyclophosphamide, epirubicin, vincristine and prednisone; CHOEP, cyclophosphamide, doxorubicin, vincristine, etoposide and prednisone; IMVP, ifosfamide, mitoxantrone, eposide; LD, lactate dehydrogenase; GC, germinal center; IPI, International Prognostic Index

### Immunohistochemical staining and sample evaluation

We used immunohistochemistry to study the expression of 8-OHdG, nitrotyrosine, Trx, MnSOD and GCL, using identical methods as in our recent work with different lymphoma entities. We have previously characterized the expression of the used antibodies in several malignant and pre-malignant tissues [18,19,22,35,36]. Sample evaluation in lymphomas has also been described earlier [22]. The cytoplasmic expression was diffuse in 8-OHdG, nitrotyrosine, Trx and GCL. MnSOD expression was granulated, in line with its previously described mitochondrial location. The nuclear staining was throughout negative in nitrotyrosine, MnSOD and GCL. 8-OHdG and Trx showed positive and diffuse staining in some of the malignant cells. The nuclear membrane was pronounced in some of the samples stained with 8-OHdG. 8-OHdG and nitrotyrosine expression in malignant cells was uniform. Trx, MnSOD and GCL showed variable intensity in malignant cell staining in few cases. These samples were coded by using the more dominant intensity. The staining results were evaluated only in malignant lymphoma cells, since in our previous work stromal staining extent or intensity did not show any clinical significance. The staining intensity of 8-OHdG, nitrotyrosine, Trx and MnSOD was divided into two groups: negative to moderate, and strong. The staining intensity of cytoplasmic GCL was divided into two groups: negative to light, and moderate to strong. We also created a combination group from Trx and nitrotyrosine immunostaining results: (i) either nitrotyrosine or Trx immunostaining intensity strong; (ii) all other immunostaining results.

### Statistical analyses

Progression-free survival was calculated from the date of diagnosis to the date of relapse or date of disease-associated death. Disease-specific survival was calculated from the date of diagnosis to the date of death caused by lymphoma. To assess the relationship between clinical parameters and oxidative stress marker expression levels we used Pearson's chi-square test. Survival estimates were calculated by using Kaplan-Meier analyses and statistical significance was determined by the log-rank test. Values of p under 0.05 were considered statistically significant. All statistical analyses were performed using the Statistical Package for the Social Sciences, v. 18.0 (Chicago, IL, USA).

## Competing interests

The authors declare that they have no competing interests.

## Authors' contributions

PP, LL and AKP participated in the design of the study, contributed to the evaluation of immunostainings and drafted the manuscript. KMH, EJ and YS helped in study design, evaluated immunostaining results, and and critically revised the manuscript. TTH contributed to study design and was also responsible for manuscript preparation. RB undertook statistical analyses. PK and OK participated in the design of the study, helped in statistical analyses and wrote the different stages of the manuscript. All authors read and approved the final manuscript.
